# Human Leukemia T-Cell Lines as Alternatives to Animal Use for Detecting Biologically Active Staphylococcal Enterotoxin Type B

**DOI:** 10.3390/toxins13050300

**Published:** 2021-04-23

**Authors:** Reuven Rasooly, Paula Do, Xiaohua He, Bradley Hernlem

**Affiliations:** Foodborne Toxin Detection & Prevention Research Unit, Western Regional Research Center, Agricultural Research Service, United States Department of Agriculture, Albany, CA 94710, USA; paula.do@ars.usda.gov (P.D.); Xiaohua.He@ars.usda.gov (X.H.); bradley.hernlem@ars.usda.gov (B.H.)

**Keywords:** staphylococcal enterotoxin type B, T-cell line, B-cell line, splenocyte

## Abstract

Staphylococcal enterotoxin type B (SEB) is associated with food poisoning. Current methods for the detection of biologically active SEB rely upon its ability to cause emesis when administered to live kittens or monkeys. This technique suffers from poor reproducibility and low sensitivity and is ethically disfavored over concerns for the welfare of laboratory animals. The data presented here show the first successful implementation of an alternative method to live animal testing that utilizes SEB super-antigenic activity to induce cytokine production for specific novel cell-based assays for quantifiable detection of active SEB. Rather than using or sacrificing live animals, we found that SEB can bind to the major histocompatibility complex (MHC) class II molecules on Raji B-cells. We presented this SEB–MHC class II complex to specific Vβ5.3 regions of the human T-cell line HPB-ALL, which led to a dose-dependent secretion of IL-2 that is capable of being quantified and can further detect 10 pg/mL of SEB. This new assay is 100,000 times more sensitive than the ex vivo murine splenocyte method that achieved a detection limit of 1 µg/mL. The data presented here also demonstrate that SEB induced proliferation in a dose-dependent manner for cells obtained by three different selection methods: by splenocyte cells containing 22% of CD4^+^ T-cells, by CD4^+^ T-cells enriched to >90% purity by negative selection methods, and by CD4^+^ T-cells enriched to >95% purity by positive selection methods. The highly enriched and positively isolated CD4^+^ T-cells with the lowest concentration of antigen-presenting cells (APC) (below 5%) provided higher cell proliferation than the splenocyte cells containing the highest concentration of APC cells.

## 1. Introduction

*Staphylococcus aureus* is a common and widespread bacterial pathogen producing twenty-three different staphylococcal enterotoxins (SEs) that are the cause of some quarter million cases of foodborne illness in the United States per year [1]. One of the most potent virulence factors generated with high ingestion and inhalation toxicity (inhalational LD_50_ ≅ 20 ng/kg of body weight) is Staphylococcal enterotoxin B (SEB), which is the only enterotoxin classified as a biological weapon due to its thermal stability, its relative ease of production, and the lack of an existing cure for it; this results in food supplies being susceptible to intentional or accidental contamination [2,3]. SEB affects the gastrointestinal system, induces vomiting, and stimulates the immune system. The immune response begins with the attachment of SEB via binding to major histocompatibility complex (MHC) class II molecules expressed on the surface of antigen-presenting cells (APC). Antigen presentation does not need antigen processing to induce T-cell activation; however, the level of cytokine secretion induced by intact superantigens was statistically lower than that for processed superantigens [4]. The toxin also binds to the variable region (V-β) on the T-cell receptor (TCR) molecule, forming a bridge between the APC and the TCR, and causes polyclonal and uncontrolled activation of CD4^+^ T-cells [2,5,6,7]. Although the super-antigenic and emetic responses are two separate effects of SEs, both responses are strongly correlated. The other studies using site-directed mutagenesis to create altered SE molecules showed an accompanying reduction in T-cell activation with the loss of emetic responses [8,9]. It was estimated that SEB was responsible for 10% of the staphylococcal enterotoxins in food poisoning outbreak cases [3,10,11]. It was shown that, among the 71 isolates originating from outbreaks of food poisoning, 54 (76.1%) possessed the SEB, SEA, SEC, or SED genes. Since those strains were capable of producing not only SEB but also SEA, SEC, and SED, it was not possible to independently evaluate the role of SEB alone in those cases of food poisoning [12]. This finding emphasized the need to develop new methods to detect biologically active SEB in order to ensure food safety and to prevent SEB from entering the food supply. The currently accepted method to detect SEs via their activity is an in vivo bioassay using live monkeys or kittens. In this bioassay, SEs are directly administered into the stomach of the animal and the animals are observed for emesis [13,14,15]. An administered dose of 10 µg of SEA produces vomiting in 50% of the animals [13,14,16]. This bioassay suffers from poor reproducibility, is expensive, has low sensitivity, and is ethically objectionable because of the requirement for the use of experimental animals, which causes concern for their welfare. Unfortunately, the sensitive immunological assays that have been developed to measure the presence of SEs cannot differentiate between the active forms of SEs (which are public health threats) and inactive toxins [15,17]. A method that can discern active toxin from inactive or inactivated toxin would be beneficial for the experimental development of treatment and for the processing operations of food. It was shown that food ingredients such as the olive compound 4-hydroxytyrosol, apple juice, and apple polyphenols inhibit biological activity in SEA [18,19]. Heat treatments, such as the pasteurization process for milk, reduced SEA activity [20], as does pulsed ultraviolet light treatment [21]. In our previous study, we utilized the superantigen activity of SEs to stimulate primary naïve CD4^+^ T-cells and developed an assay that can distinguish between active and inactive toxins in food [20,22,23]. These methods utilize the induction by SEs of cytokines including IL-2 [22,23], IFN-γ [24], TNF, [25], and the cell surface marker CD154 [26]. We measured SEs by the quantification of cytokine secretion using flow cytometry [4,26] or qRT-PCR [24]. However, to eliminate any use of animals in our assays, we utilized a human T-lymphocyte Jurkat cell-line expressing the luciferase reporter gene and developed a bioluminescence-based assay for the detection of biologically active SEE and SED [23,27]. We also demonstrated that SEE specifically causes the internalization of TCR Vβ8 in a dose-dependent manner and developed a flow cytometric assay for the detection and quantitation of biologically active SEE [28]. Correspondingly, we demonstrated that active SEA specifically reduces surface TCR Vβ9 in a dose-dependent manner and used this observation to quantify the biologically active SEA [4]. However, those assays cannot detect SEB. The objective of this study was then to develop alternative methods to in vivo monkey or kitten bioassays for SEB detection that would be able to differentiate between active and inactive SEB. 

## 2. Results

### 2.1. Ex Vivo Cell-Based Assay

**Figure 1:**
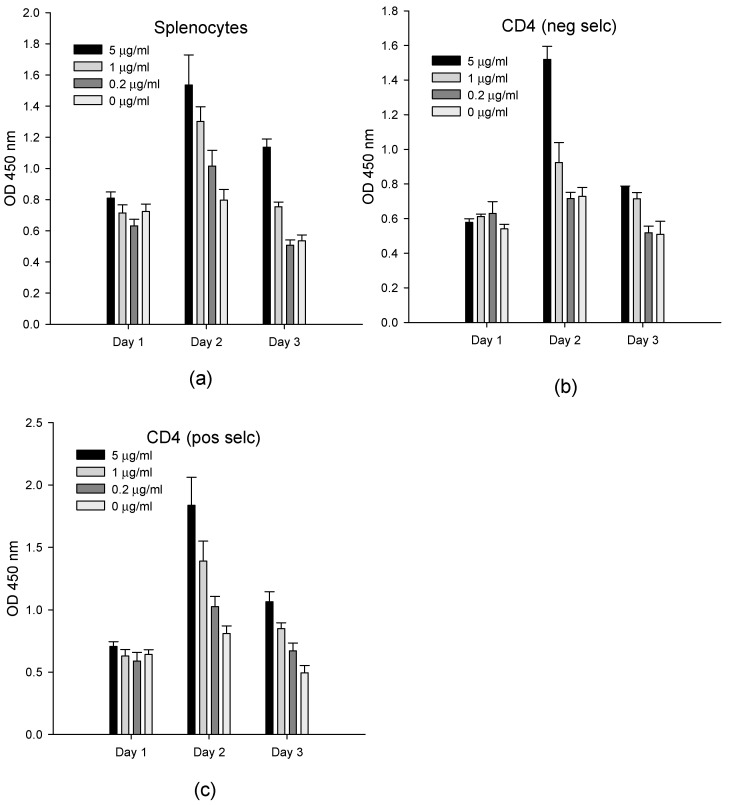
The SEB activation of splenocytes and purified CD4^+^ T-cells. Splenocytes (**a**), negatively Scheme 4. T-cells (**b**), and positively selected CD4^+^ T-cells (**c**) were incubated with 5, 1, and 0.2 µg/mL of SEB. After incubation for one to three days, the newly synthesized DNA was measured on each day. The error bars represent standard errors.

In order to evaluate the ability of the ex vivo assay to quantify SEB, we measured the effect of SEB at concentrations of 5, 1, and 0.2 µg/mL on splenocyte cells, which contained 22% of CD4^+^ T-cells on a population that was enriched by the negative selection of CD4^+^ T-cells with >90% purity and on a population enriched by the positive selection of CD4^+^ T-cells with >95% purity (Figure 1). After one to three days, the BrdU stain was added to cell cultures, and then, the T-cell proliferation was spectroscopically measured. Our result shows that we are unable to detect any activity of cell proliferation during the first 24 h of incubation with SEB. However, at both time periods of 48 h and 72 h, the amount of DNA synthesized in the cells since the commencement of the experiment was linearly correlated with the SEB concentration in splenocytes and with positively and negatively selected purified CD4^+^ T-cells. The highly enriched and positively isolated CD4^+^ T-cell population, which had the lowest fraction of APC cells (below 5%), provided a higher signal than the splenocyte cells containing the highest concentrations of APC cells. The limit of detection was 1 µg/mL of SEB in both the splenocytes and CD4^+^ T-cells. T-cell proliferation peaked at 48 h and was reduced after 72 h in all three cell types.

### 2.2. In Vitro Cell-Based Assay

**Figure 2:**
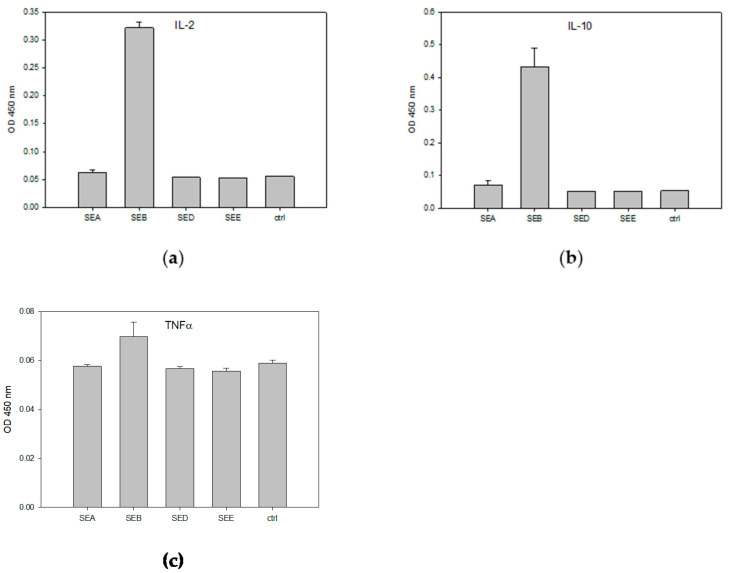
Assay cross-reactivity and specificity. HPB-ALL T-cells and Raji B-cells were incubated at 48 h with 1 µg/mL of SEA, SEB, SED, SEE, or control media. The specific inductions of IL-2 (**a**), IL-10 (**b**), and TNF-α (**c**) secretion were measured by ELISA. The error bars represent standard errors.

The ex vivo method described in the previous section has further advantages over the in vivo bioassays that used monkeys or kittens. Far fewer animals are required because a single mouse spleen can provide enough cells for as many as 500 tests. However, this ex vivo bioassay is still ethically problematic because of the necessary sacrifice of live experimental mice. Consequently, we looked for suitable cell lines that could be used as an alternative to mouse splenocytes and conducted experiments evaluating the use of the human T-cell acute lymphoblastic leukemia cell line (HPB-ALL) for the detection and quantification of biologically active SEB. We utilized Raji B-cells as antigen-presenting cells (APCs) in conjunction with HPB-ALL. The combined cultures of HPB-ALL and Raji cells were then incubated for 48 h with various concentrations of SEB. The relative concentrations of interleukin 2 (IL-2), interleukin 10 (IL-10), and tumor necrosis factor alpha (TNF-α) secreted by HPB-ALL and Raji B-cells were determined using ELISA. Since SEB shares considerable similarity in terms of amino acid sequence with other SEs, we applied SEA, SED, and SEE to evaluate the cross reactivity and the specificity of the assay. One-way analysis of variance (ANOVA) was then used to identify any statistically significant differences between the various cell treatments and control. These data are presented in Figure 2.

### 2.3. HPB-ALL Cells Express Vβ5.3

**Figure 3:**
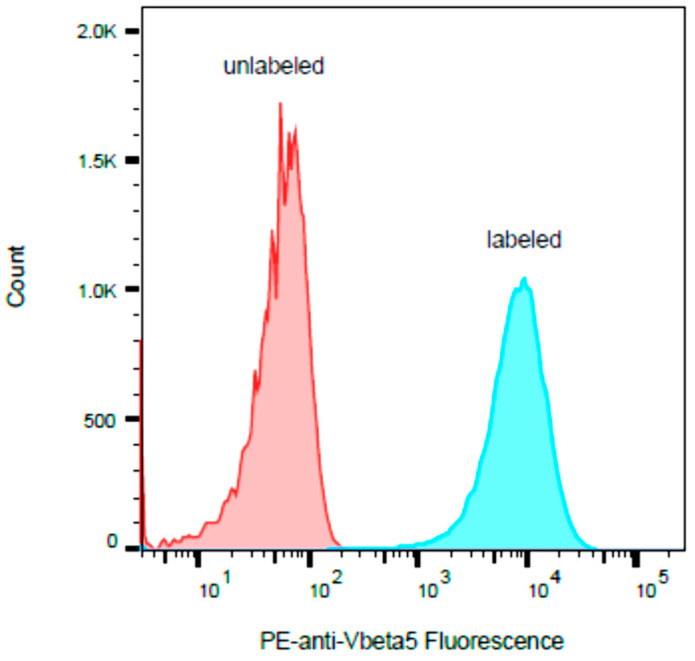
The human HPB-ALL T-cell line was stained with PE-labeled antibody to Vβ5.3 and compared with unstained cells. The histogram *y*-axis represents the relative number of cells, and the histogram *x*-axis represents the cell-associated PE fluorescence on a logarithmic scale.

It has been reported that SEB stimulates human T-cells that bear any of the Vβ subunit variants 1.1, 3.2, 6.4, 12, 13.2, 14, 15.1, 17, 20, and 22 and that SEA stimulates human T-cells that bear Vβ5.3 [29]. Our flow cytometric analysis in Figure 3 shows that the human HPB-ALL T-cell line expresses the Vβ5.3 gene.

There were unexpected associations found between SEB and Vβ5.3. As shown in the ELISA results in Figure 2, SEB at a concentration of 1 µg/mL stimulates human HPB-ALL T-cells and induces the secretion of levels of IL-2, IL-10, and TNF-α above that of the control and without cross reactivity with SEA, or SED and SEE. This activity assay is highly specific for the detection of SEB, and only SEB was able to induce the secretion of IL-2, IL-10, and TNF-α using this pair of cell lines. However, the latter two cytokines are not complimentary, and although TNF-α secretion was found to be stimulated by SEB compared to the control and the other SEs tested, the anti-inflammatory cytokine IL-10 was reported to block the production of TNF-α and to reduce the rate of lethal superantigen-induced toxic shock [30]. The lower degree of stimulated TNF-α secretion may be a result of the co-secretion of IL-10.

### 2.4. Limit of Detection

**Figure 4:**
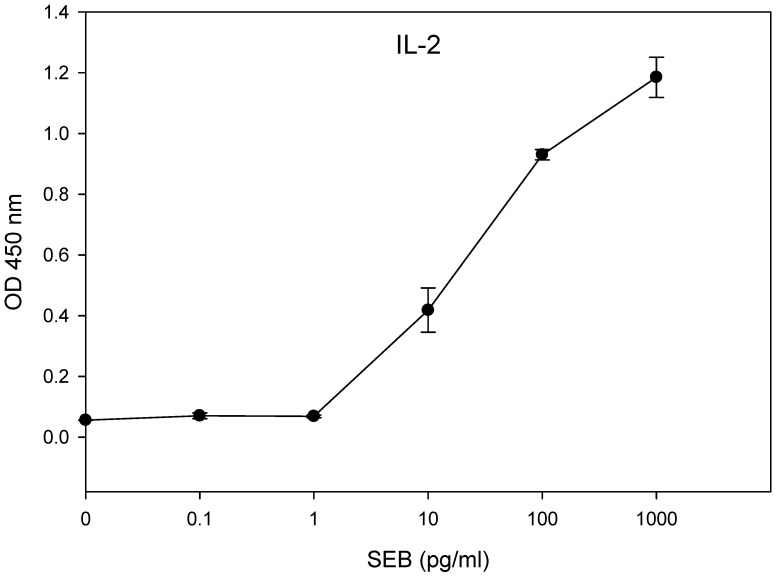
In vitro assay for the quantification of biologically active SEB. The HPB-ALL T-cells in a co-culture with Raji B-cells were incubated in a 96-well plate for 48 h with increasing concentrations of SEB. The IL-2 concentration was measured by ELISA using a microplate reader with a 450 nm filter. The error bars represent standard errors.

The limit of detection for the presence of biologically active SEB was determined using concentrations of SEB ranging from 1 mg/mL to 0.1 pg/mL in the 48-hour incubation of HPB-ALL T-cells in combination with the Raji B-cells. The results in Figure 4 show that the amount of secretion of IL-2 was significantly different (*p* < 0.05) between the treatment and the control. We observed a limit of detection of 10 pg/mL of SEB. 

## 3. Discussion

In this research study, we evaluated a specific assay for SEB using a cell-based alternative method to the emetic bioassays using monkeys or kittens, which are the currently accepted methods used to measure the biological activity of Staphylococcal enterotoxins. We present, for the first time, successful use of the human T-cell acute lymphoblastic leukemia (HPB-ALL) cell line in combination with a B-cell line, leading to the development of an inexpensive alternative to live animal testing for the detection and quantification of biologically active SEB. The essential mechanism for this assay is complexation of the SEB toxin and the MHC class II molecules expressed on the surface of Raji B-cells, which then bind to the T-cell receptors of HPB-ALL cells, leading to super-antigenic activation. The result is a new assay that is 100,000 times more sensitive than the ex vivo murine splenocyte-proliferative response. SEB is a threat to public health with respect to accidental contamination or poor handling, and it is also classified as a potential biological weapon with respect to the intentional contamination of food supplies. It was reported that SEA, but not SEB, stimulates human T-cells bearing Vβ5.3 [29]. However, our ELISA results in Figure 3 and Figure 4 show that SEB stimulates human HPB-ALL T-cells and induces the secretion of higher levels of IL-2, IL-10, and TNF-α than the control. Despite SEB having considerable amino acid sequence similarity with the other common SEs, this cell-based assay is specific to SEB and has no cross reactivity with SEA, SED, and SEE (Figure 3). It is possible that the T-cell receptor that is expressed on the surface of the HPB-ALL cell line contributes to the assay specificity. This preference between specific T-cell lines and specific SE subtypes has been noted before. When we applied SEA, SEB, SED, and SEE to the CCRF-CEM T-cell line in combination with the Raji B-cell line, the results show that this assay is very specific to SEA detection; only SEA activated the CCRF-CEM T-cell line [31]. Prior research has shown the relationship between super-antigenic activity and the emetic activity of Staphylococcal enterotoxin. The antibodies that emerged as a result of immune response against a nontoxic mutant of SEA were found to inhibit both the T-cell proliferation induced by toxic SEA and the emetic activity of the active toxin [8]. The site-directed mutagenesis applied to eliminate the emetic activity of Staphylococcal enterotoxin type C2 further resulted in the elimination of SEC2 super-antigenic activities [9]. It was, therefore, assumed that an assay capable of measuring super-antigenic activity will likewise be capable of predicting emetic response and be capable of discerning active enterotoxin. The ability to differentiate between the inactive and active forms of enterotoxin, which poses a threat to public health, is important for the development of effective food treatment methods. Heat treatment, such as the pasteurization process for milk, reduced SEA activity [20] and pulsed ultraviolet light treatment [21]. The results of this research show that highly enriched and positively isolated murine CD4^+^ T-cells with less than 5% prevalence of APC provided higher cell proliferation by stimulation with SEB than murine splenocyte cells containing a high natural concentration of APC cells. This appears to suggest that the requirement for the interaction of two separate types of cells, the T-cell and an accessory, as a safety mechanism in immune response does not exist in the response to SEB. Prior research has shown that treatments utilizing the neutralizing anti-MHC class II antibody blocks the proliferation of purified CD4^+^ T-cells in response to SEA [32]. The data in that study suggest that a subpopulation of purified CD4^+^ T-cells, by the presentation of an SE molecule via MHC class II, can perform both the roles of the traditional APCs and of T-cells, providing adequate accessory signals to themselves or proximate CD4^+^ T-cells to trigger T-cell proliferation. 

## 4. Materials and Methods

### 4.1. Chemicals and Reagents

The SEB, SEA, SED, and SEE toxins were obtained from Toxin Technology (Sarasota, FL, USA), and their purity levels at >95% were determined by SDS-PAGE and the Coomassie blue stain. The toxins were reconstituted in water. The PE-conjugated mouse anti-human Vβ5.3 antibody was obtained from Novus Biologicals (Centennial, CO, USA).

### 4.2. Media

The cell culture media for splenocytes were sourced from Gibco (Gibco/Thermo Fisher, Waltham, MA, USA). The cell growth medium consisted of RPMI-1640 with the addition of 10% fetal bovine serum (HyClone, Logan, UT, USA), 200 mM of glutamine, 1 mM of sodium pyruvate, 1× MEM (Roswell Park Memorial Institute medium) antibiotic-antimycotic, and nonessential amino acids. For murine splenocyte cells, we added an additional 50 µM of beta-mercaptoethanol to the media (Sigma, St. Louis, MO, USA). The Russ-10 cell culture medium consisted of 450 mL of RPMI-1640 medium without glutamine (Gibco), 10% fetal bovine serum (HyClone, Logan, UT, USA), 5 mL of sodium pyruvate (Gibco), 0.25 mL of 100 mM β-mercaptoethanol (Sigma), 5 mL of 200 mM glutamine (Gibco), 5 mL of antibiotic-antimycotic (Gibco; containing penicillin, streptomycin, and fungizone), and 5 mL of nonessential amino acid mix (Gibco). The lysis buffer consisted of 150 mM of NH_4_Cl, 100 µM of Na_2_EDTA, and 10 mM of KHCO_3_. 

The cell culture media for the human cell lines were comprised of RPMI 1640 supplemented with 10% FCS, 1% MEM nonessential amino acids, 100 nM of sodium pyruvate, and antibiotic-antimycotic solution. The cell cultures were maintained under a humidified atmosphere containing 5% CO_2_ in an incubator kept at 37 ℃.

### 4.3. Splenocyte Isolation and Human Cell Lines 

The spleens from female C57BL/6 mice were aseptically removed, and a syringe with a needle filled with the culture medium was used to harvest the splenocytes. The suspension of cells was then centrifuged at 200× *g* at 4 °C for 10 min. The red blood cells were lysed with a lysis buffer and then centrifuged and resuspended in the Russ-10 medium. The viable cells were counted using trypan blue and a hemocytometer. 

The human T lymphoblastoid line HPB-ALL and the Raji B-cell line were obtained from the Leibniz Institute DSMZ-German Collection of Microorganisms and Cell Cultures (Germany). All cells were maintained in an incubator at 37 °C under a humidified 5% CO_2_ atmosphere. 

### 4.4. Positive or Negative Isolation of Murine CD4+ T-Cells

For the positive and negative isolations of murine CD4+ T-cells, the Dynabeads Mouse CD4 L3T4 Positive Isolation kit and the Dynal Mouse CD4 Negative Isolation Kit were used according to the manufacturer’s instructions (Thermo Fisher Scientific). For the positive selection isolation buffer, PBS supplemented with 0.1% BSA and 2 mM of EDTA was briefly used to resuspend splenocytes at a concentration of 1 × 10^7^/mL. The Dynabeads (25 µL of Dynabeads per 10^7^ cells) were washed and incubated with splenocytes for 20 min on ice and were gently rotated. Afterwards, the cells containing the Dynabeads were placed on a magnet for 2 min. The media were then removed, the cells still attached to the Dynabeads were washed three times with the isolation buffer, and then they were resuspended in Russ-10 media (10^7^ cells per 100 µL of media). The DETACHaBEAD mouse CD4 was added (10 µL per 10^7^ cells) and incubated for 45 min with gentle rotation at room temperature. The cells detached from the Dynabeads were then washed three times and resuspended in media. For the negative selection of CD4 cells, heat-inactivated FBS and antibody were added to the splenocytes and incubated for 20 min on ice. The cells were washed with an isolation buffer, and pre-washed mouse depletion Dynabeads were then added and incubated for 15 min with gentle rotation at room temperature. The cells and the Dynabeads were then placed on a magnet, and the supernatant was obtained and further washed. The supernatant contained the negatively isolated mouse CD4 T cells.

### 4.5. Quantitative Determinations of SEs by Cytokine Secretion

In a clear 96 well plate, 50 µL of 2 × 10^6^ cells per mL suspension of HPB-ALL cells, 25 µL of a 2 × 10^6^ cells per mL suspension of Raji cells, and 25 µL of SEs at four times the final target concentration were combined in a cell culture medium. The cells were then incubated at 37 °C for up to two days. The supernatants were then harvested after 24 and 48 h and were further tested for cytokine secretion (IL-2, IL-10, and TNFα) by ELISA following the manufacturer’s instructions (BD Bioscience OptEIA Human ELISA).

### 4.6. Measurement of Bromodeoxyuridine (BrdU)

After incubation, cell proliferation was measured by adding bromodeoxyuridine (5-bromo-2-deoxyuridine, BrdU)-labeled DNA to each well 4 h before fixation, as instructed by the outlines from the manufacturer (Calbiochem, San Diego, CA, USA). We briefly describe this procedure: diluted BrdU label was added to the cells and incubated at 37 °C for 4 h. The labeling medium was then removed by spinning the cells at 200× *g* for 10 min. Fixative denature reagent (200 µL/well) was added and incubated for 30 min at RT and then decanted. The anti-BrdU antibody, attached to horse radish peroxidase, was then added (100 µL/well) and incubated for 90 min at RT. The wells were washed three times, the substrates were added at 100 µL/well, and then they were incubated until development was sufficient (5–30 min). The spectroscopic measurements were made at absorbances of 620 and 450 nm.

### 4.7. Flow Cytometry

Flow cytometry was performed using a FACSAria Fusion instrument from BD Biosciences (San Jose, CA, USA). The data analysis was performed using FlowJo software from BD Biosciences.

### 4.8. Statistical Analysis

Statistical analysis was performed with SigmaStat 3.5 for Windows (Systat Software, San Jose, CA, USA). One-way analysis of variance was used to determine the detection of SEB. The experiments were repeated at least three times, and only results with *p* < 0.05 were considered statistically significant. A *t*-test analysis was used to determine whether there were any significant differences between treatment and the control.

### 4.9. Ethics Statement

All of the procedures with animals were carried out according to institutional guidelines for husbandry approved by the Institutional Animal Care and Use Committee of the U.S. Department of Agriculture, Western Regional Research Center (USDA IACUC). These specific procedures and protocols were reviewed and approved by the USDA IACUC (Protocol #13-1). The mice were euthanized using rapid cervical dislocation to minimize their suffering.

## Data Availability

The data presented in this study are available upon request from the corresponding author.
